# Gastrodin protects dopaminergic neurons via insulin-like pathway in a Parkinson’s disease model

**DOI:** 10.1186/s12868-019-0512-x

**Published:** 2019-06-17

**Authors:** Jinyuan Yan, Zhongshan Yang, Ninghui Zhao, Zhiwei Li, Xia Cao

**Affiliations:** 1 The Second Affiliated Hospital of Kunming Medical University, 374 Dian Mian Road, Kunming, 650101 Yunnan China; 2 Faculty of Basic Medicine, Yunnan University of Chinese Medicine, Kunming, 650500 Yunnan China

**Keywords:** Gastrodin, Dopamine neurons, α-Synuclein, DAF-2/DAF-16 pathway, Parkinson’s disease

## Abstract

**Background:**

Recently, the use of traditional Chinese medicine (TCM) has become more generally accepted, including by the Food and Drug Administration. To expand the use of TCM worldwide, it is important to study the molecular mechanisms by which TCM and its active ingredients produce effects. Gastrodin is an active ingredient from *Gastrodia elata Blume*. It is reported that gastrodin has neuroprotective function in Parkinson’s disease. But its mechanisms of neuroprotection remain not clear in PD. Here, we build two *C. elegans* PD model using 6-OHDA and transgenic animal to observe the changes of PD worms treated with or without gastrodin to confirm the function of gastrodin, then utilize mutant worms to investigate DAF-2/DAF-16 signaling pathway, and finally verify the mechanism of gastrodin in PD.

**Results:**

Gastrodin attenuates the accumulation of α-synuclein and the injury of dopaminergic neurons, improves chemotaxis behavior in Parkinson’s disease models, then recovers chemotaxis behavior by insulin-like pathway. DAF-2/DAF-16 is required for neuroprotective effect of dopamine neuron in PD.

**Conclusions:**

Our study demonstrated that gastrodin rescued dopaminergic neurons and reduced accumulation of α-synuclein protein, and the activity of gastrodin against Parkinson’s disease depended on the insulin-like DAF-2/DAF-16 signaling pathway. Our findings revealed that this insulin-like pathway mediates neuroprotection of gastrodin in a Parkinson’s disease model.

**Electronic supplementary material:**

The online version of this article (10.1186/s12868-019-0512-x) contains supplementary material, which is available to authorized users.

## Background

The incidence and prevalence of Parkinson’s disease (PD) continue to increase worldwide [1, 2]. Parkinson’s disease is a common disease in older people, and is the second main neurodegenerative disease worldwide. The symptoms of PD include bradykinesia, rigidity, abnormal posture, resting tremors, olfactory dysfunction, and cognitive disorder. PD is characterized by a loss of 80% of substantia nigra dopamine (DA) neurons and the excess accumulation of α-synuclein protein [3, 4]. Genetic factors, environmental factors, aging, toxin, infection, and potentially other factors may cause PD. About 10% of PD cases are inherited forms of disease, and many PD-associated genes have been identified, including SNCA(PARK1/4), Parkin(PARK2), LRRK2(PARK8), PINK1(PARK6), UCHL-1(PARK5), DJ-1(PARK7), ATXN2, ATXN3, PLA2G6, ATP13A2(PARK9) and so on [5, 6]. Currently, levodopa is widely used clinically, countering PD symptoms by maintaining normal dopamine metabolism. However, levodopa only alleviates symptoms, and does not alter the development of PD. Additionally, the long-term use of levodopa may cause side-effects, such as involuntary movements, agitation, clumsiness, low blood pressure, slowed gastrointestinal peristalsis, and even mental symptoms [7]. Overall, there remains a lack of effective drugs and treatments to both treat symptoms and delay the onset of disease. Hence, finding a medicine with neuroprotective effects would be highly valuable for improved treatment of PD.

Gastrodin is an active ingredient of the orchid plant *Gastrodia elata Blume*, and is a commonly used traditional Chinese medicine. Gastrodin is reported to have antioxidant, anticonvulsant, anti-inflammatory, analgesic, sedative, and anxiolytic properties, and can improve learning and memory [8–12]. Gastrodin reduces mRNA and protein levels of inflammatory enzymes and proinflammatory cytokines that are induced by the suppression of NF-kB signalling and the phosphorylation of mitogen-activated protein kinases (MAPK) in microglial cells induced by lipopolysaccharide [9]. Gastrodin can cross the blood–brain barrier, and then is decomposed into p-hydroxy benzyl alcohol in the central nervous system, blood, and liver to protect the nervous system from damage [13]. In 6-OHDA rat model of PD, gastrodin with microinjection in intra-cerebro ventricular lowers myeloperoxidase, the levels of lipid peroxidation and NO production, rescues antioxidant capacity levels, and ameliorates motor incoordination of rats [14]. In MPP^+^ induced SH-SY5Y model of PD, gastrodin combination with isorhynchophylline regulates ERK1/2 and GSK-3β pathways, increases Nrf2 nuclear accumulation to decrease oxidative stress for neuroprotective effect [15]. Kumar et al. reports that gastrodin has an anti-apoptosis role in PD model [16]. The study of cellular PD models also suggest that gastrodin counteract oxidative stress and p38 MAPK/Nrf2 pathway to protect dopamine neurons [17]. Although gastrodin is a potential drug for the treatment of neurodegenerative diseases, its mechanisms of neuroprotection remain unclear. Hence, the pathway through which gastrodin mediates neuroprotective effect in PD should be elucidated to better understand its function and potentially expand its use for disease treatment.

In our study, we selected the *Caenorhabditis elegans* (*C. elegans*) model to study PD. *C. elegans* is a robust animal model with many advantages because it is of small size, has a short life cycle, is inexpensive, and multiple transgenic strains exist, enabling the study of various human diseases. Here, we demonstrated that gastrodin alleviated dopamine neuron injury and α-synuclein protein aggregation, and regulated the DAF-2/DAF-16 insulin-like signaling pathway to decrease dopaminergic neurons degeneration in a *C. elegans* model of Parkinson disease.

## Results

### Gastrodin attenuates the accumulation of α-synuclein and the injury of dopaminergic neurons in different Parkinson models

**Fig. 1:**
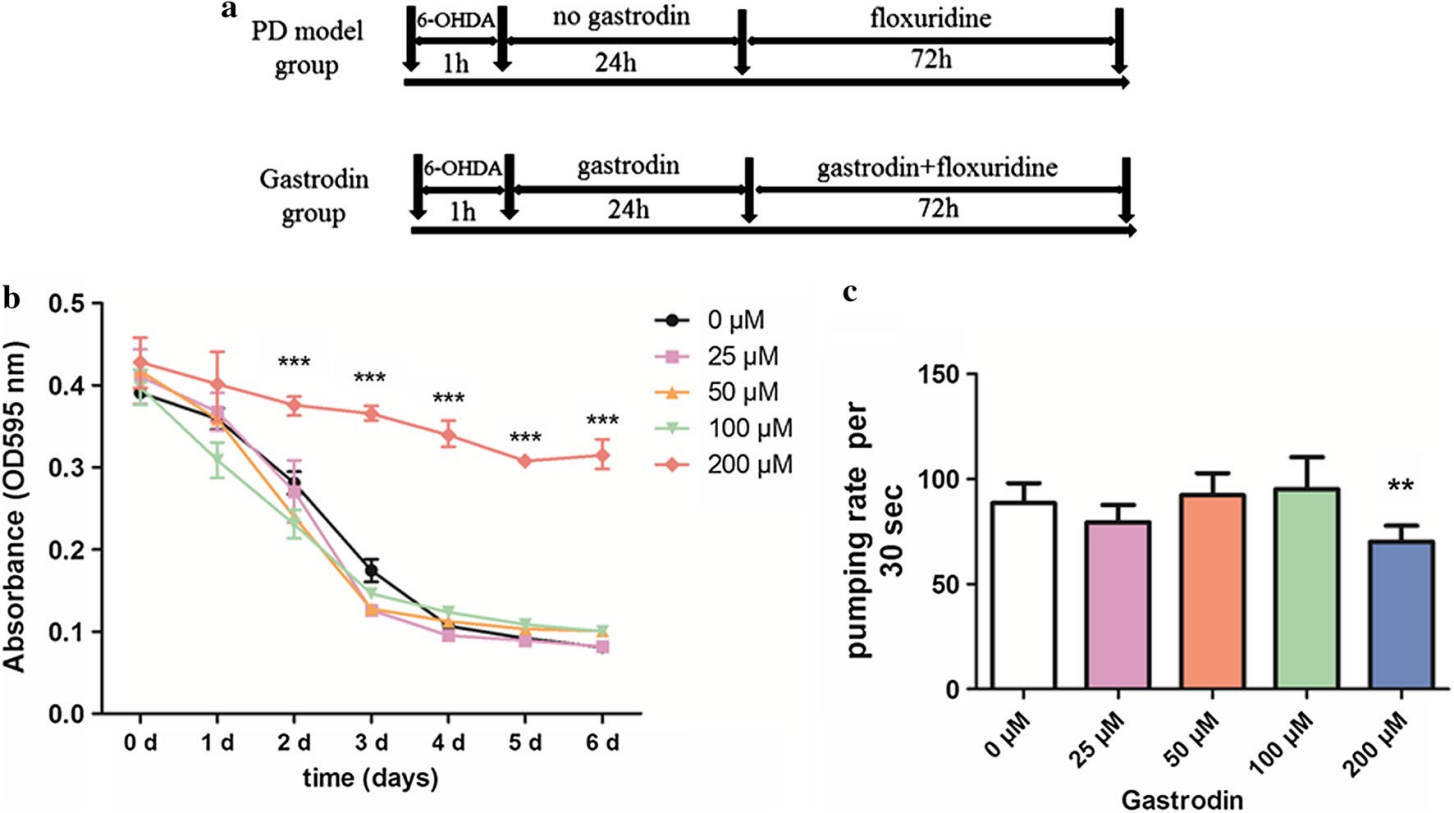
Determination of the appropriate assay concentration of gastrodin. **a** The schematic diagram of the timeline about building PD model and gastrodin treatment. **b** The food clearance rates of worms for different concentrations of gastrodin were determined. The statistical differences were analyzed using two-way RM ANOVA following by Bonferroni post-tests to compare replicate means by row (**c**) Gastrodin at 200 μM obviously reduced the pumping rates of worms. The statistical differences were analyzed using one-way ANOVA. ns *P* ≥ 0.05, *P < 0.05; **P < 0.01; ***P < 0.001

The function of gastrodin in the treatment of neurodegenerative disease has been widely reported, but its mechanism of action is poorly clarified. To determine whether gastrodin affected the normal physiological function of animals, we chose the *C. elegans* PD model. We first investigated the food clearance and pumping rate (ingestion rate) assay in wild type animals fed 0, 25, 50, 100, or 200 μM gastrodin. The food clearance and pumping rate assay was supplied in this section for detecting the effect of gastrodin on *C. elegans* physiology and monitoring the right concentration [18–20]. As shown in Fig. 1b, the food clearance of animals with 200 μM gastrodin was significantly reduced than that of worms in the absence of gastrodin from 2 to 6 days (*P* < 0.001, *F* = 58.87. *df* = 4, two-way RM ANOVA following by Bonferroni post-tests). We also observed a lower pumping rate for animals fed 200 μM gastrodin compared to no gastrodin group (*P* = 0.0077 < 0.01, *F* = 3.96, *df* = 4, 45) (Fig. 1c). Hence, we used concentrations of 25, 50, and 100 μM gastrodin for the following assays.

**Fig. 2:**
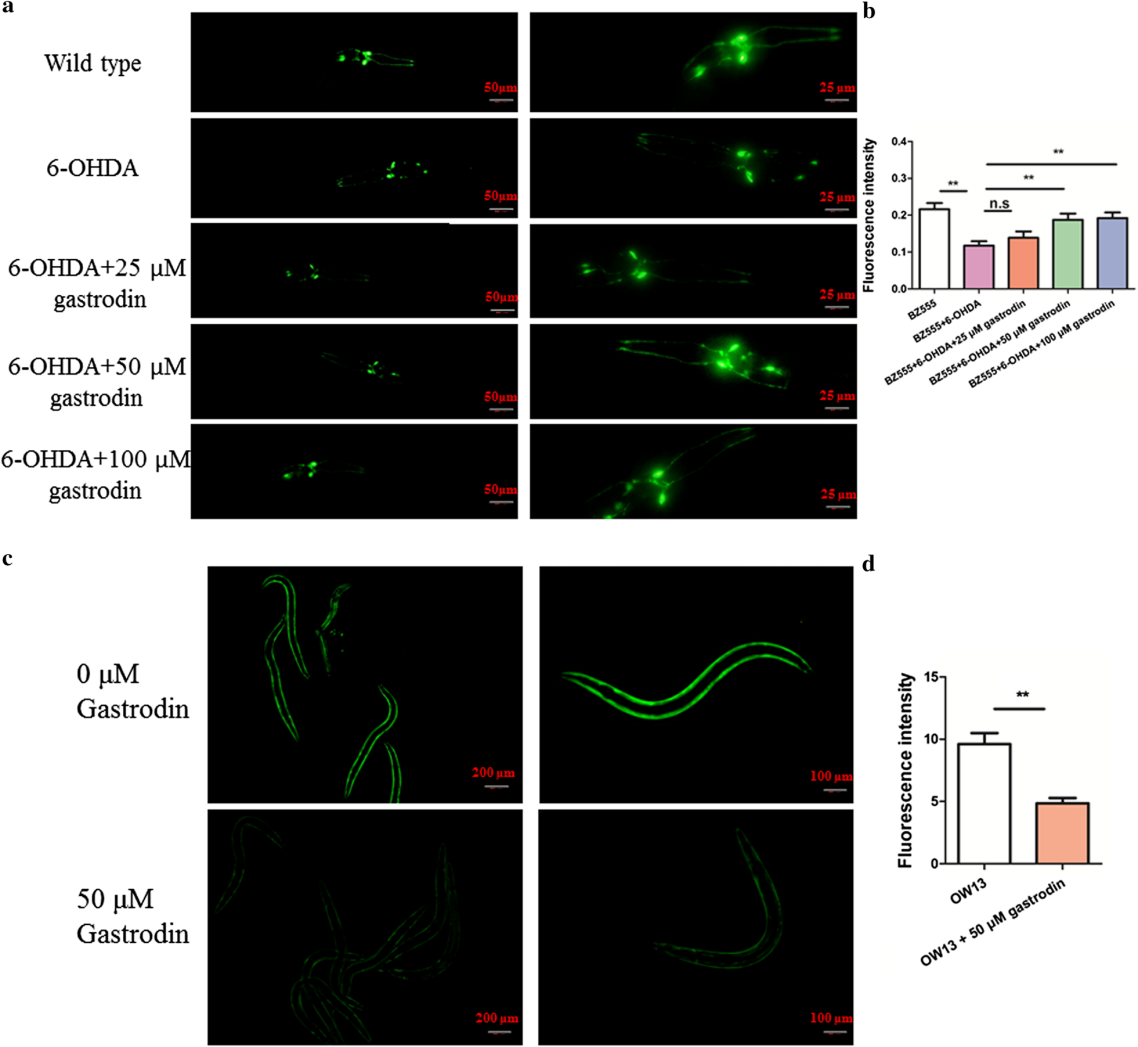
Gastrodin can alleviate the injury of dopamine neurons and accumulated α-synuclein in two PD models. **a** Both 50 and 100 μM gastrodin restored the loss of GFP of dopaminergic neurons with 6-OHDA treatment. **b** Quantification of dopaminergic neurons in *C. elegans* using Image J software by one-way ANOVA. (The scale bar and magnification of left panel is 50 μm, 200; The scale bar and magnification of right panel is 25 μm, 400, respectively). **c** Gastrodin markedly lessened GFP of α-synuclein in the PD model with overexpressed α-synuclein. **d** The fluorescence intensity of α-synuclein was also analyzed by Image J. (The scale bar and magnification of left panel is 200 μm, 40; The scale bar and magnification of right panel is 100 μm, 100). The used statistical differences were *t*-test. ns *P* ≥ 0.05; *P < 0.05; **P < 0.01; ***P < 0.001

We next evaluated the effect of gastrodin (25, 50, and 100 μM) on two Parkinson models, one produced by 6-OHDA treatment and one produced by transgenic worm overexpression of α-synuclein. After 6-OHDA treatment, dopamine neurons and axons marked with BZ555 [*egIs1 [dat*-*1p::GFP]*] were impaired (Fig. 2a). However, the expression of *Pdat*-*1::gfp* in the 6-OHDA induced model with addition of 50 and 100 μM gastrodin showed obviously higher than that of 6-OHDA PD model (*P* = 0.0030, 0.0067 < 0.01; *F* = 1.592. 2.719; *df* = 6, 6; Fig. 2a, b). A lesser effect was seen for higher amounts of 25 μM (*P* = 0.0940 > 0.05, *F* = 2.880, *df* = 6), suggesting 50 μM gastrodin was required for better protection in the Parkinson model. At 50 μM gastrodin in OP50/NGM plates, the accumulation of α-synuclein in transgenic strain OW13 [*grk*-*1(ok1239) X; pkIs2386 IV*] overexpressing the human α-synuclein protein showed a remarkable decrease (*P* = 0.0086 < 0.01, *F* = 4.49, *df* = 4; Fig. 2c, d). Taken together, these data suggest that the addition of 50 μM gastrodin had an obvious effect on two parameters of PD disease.

### Gastrodin improves chemotaxis in PD animals

**Fig. 3:**
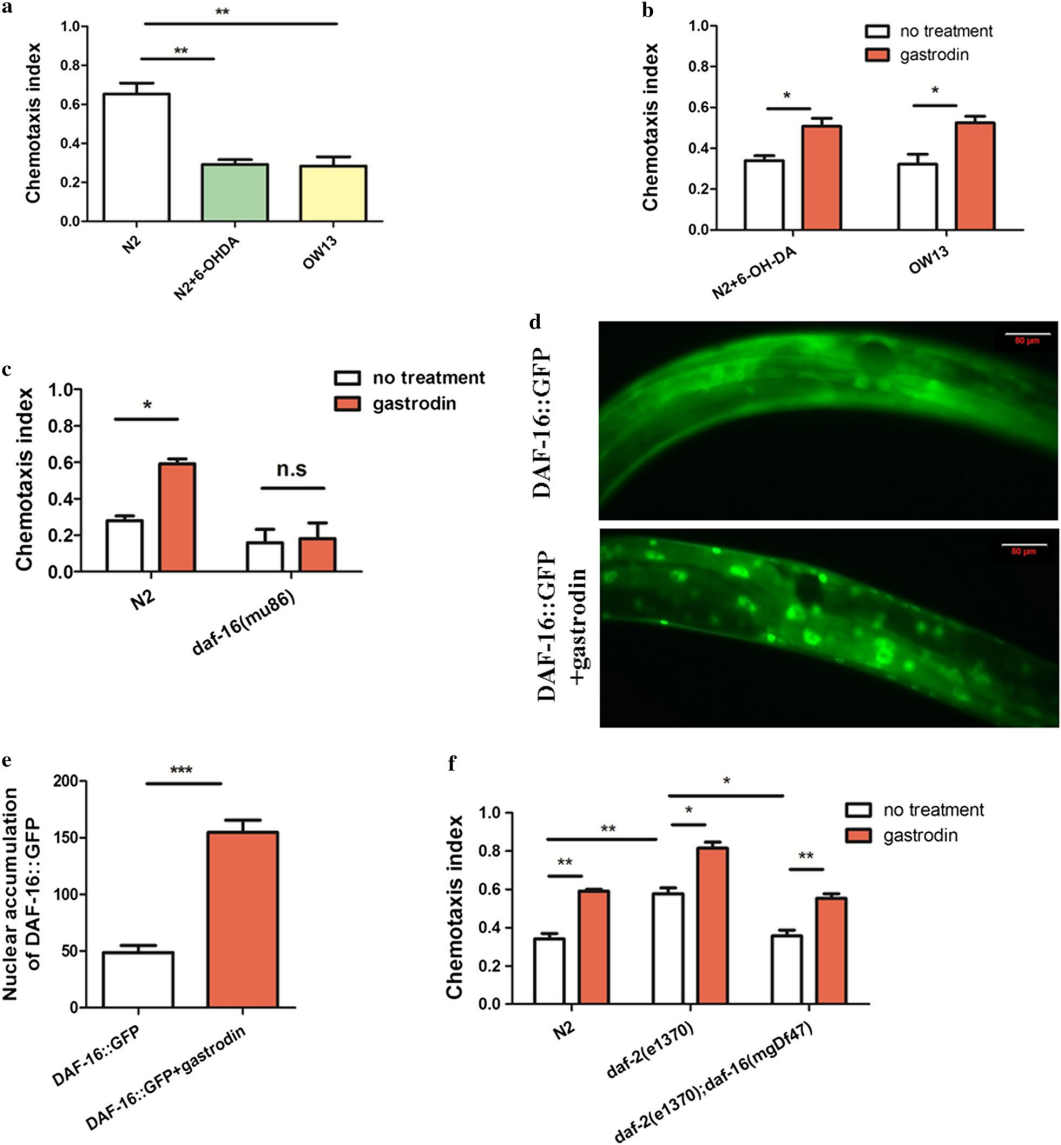
Gastrodin rescues chemotaxis behavior by DAF-2/DAF-16. **a** In the two PD models, the chemotaxis behavior of worms was increased after treatment with gastrodin. **b** Gastrodin obviously increased chemotaxis behavior in two PD models. **c** The mutant *daf*-*16(mu86)* shows decreased chemotaxis compared to wild type, and treatment of the mutant with gastrodin did not rescue the behavior. **d** Gastrodin increased the nuclear accumulation of DAF-16::GFP. The scale bar and magnification of left panel is 50 μm, 200. **e** Quantification of DAF-16::GFP nuclear accumulation in no gastrodin and gastrodin conditions. **f** *daf*-*2* mutation exhibited an increased chemotaxis index, and the double-mutants *daf*-*2;daf*-*16* restored the level to that of wild type animals. The statistical differences were analyzed using *t*-test method, ns *P* ≥ 0.05; *P < 0.05; **P < 0.01; ***P < 0.001

The loss of dopamine neurons can affect olfactory transmissions and olfactory bulb function [21–24]. About 90% of patients with Parkinson’s disease exhibit olfactory dysfunction symptoms [24]. Aging, α-synuclein accumulation, inflammation response, tau deposition, dopamine loss, and environmental elements may contribute to olfactory deficits in PD [24]. Hence, olfactory dysfunction may serve as a sensitive marker of PD [25]. Thus, we measured olfactory function in the PD model by observing the attractive behavior/chemotaxis behavior to the volatile compounds 2-heptanone and benzaldehyde. A significant decrease in chemotaxis to 2-heptanone in both 6-OHDA-treated and OW13 worms was observed relative to the chemotaxis behavior of wild type worms (*P* = 0.0041, 0.0073 < 0.01; *F* = 5.384, 1.420; *df* = 4, 4; Fig. 3a), but treatment with gastrodin increased the chemotaxis index for the deficient worms (*P* = 0.0215, 0.0263 < 0.05; *F* = 2.566, 2.401; *df* = 4, 4; Fig. 3b). This result indicated that chemotaxis can be used as an indicator of PD in further experiments. In addition, expression of BZ555, as indicated by GFP signal, was not changed after treatment with gastrodin relative to the control (*P* = 0.9798 > 0.05, *F* = 1.167, *df* = 6; Additional file 1: Fig. S1). Overall, we conclude that 6-OHDA-treated and OW13 transgenic worms show deficient olfactory function as evidenced by altered chemotaxis behavior to volatiles.

### Gastrodin recovers chemotaxis behavior by insulin-like pathway

Accumulating evidences suggest that insulin-like pathway may be involved in neurodegenerative diseases [26–28]. In *C. elegans,* insulin-like signaling mainly contains insulin-like receptor DAF-2 and forkhead transcription factor DAF-16 which is negatively regulated by DAF-2 [29]. After DAF-2 is inactive, DAF-16 nuclear is translocated from the cytoplasm to nuclei. Then, activation DAF-16 regulates stress response, life span, and metabolism. Since the activation of DAF-16 (mammalian homologue Foxo3) in *C. elegans* can extend lifespan, and resist stress and infection [30–32], we hypothesized that gastrodin might mediate neuroprotection by activation of DAF-16. We first determined the chemotaxis behavior of the *daf*-16 mutant. We found that knockdown of *daf*-*16* resulted in reduced olfactory function (Fig. 3c). Then, we found that gastrodin could obviously increase content of dopamine in wild type animals (*P* = 0.0013 < 0.01, *F* = 3.524, *df* = 4), and the dopamine in *daf*-*16(mu86)* mutant was reduced by LC–MS/MS method (*P* = 0.7565 > 0.05, *F* = 1.089, *df* = 4) (Additional file 2: Fig. S2). We then treated the *daf*-*16* mutant with gastrodin. If gastrodin mediated neuroprotection through activation of DAF-16, then we would expect that gastrodin treatment would not be able to improve the deficient chemotaxis behavior of the *daf*-16 mutant. Consistent with this model, no response to gastrodin was observed (*P* = 0.8514 > 0.05, *F* = 1.376, *df* = 4; Fig. 3c).

It is known that DAF-16 activation is controlled by nuclear accumulation [33–35]. We next asked if gastrodin could induce nuclear accumulation of DAF-16:: GFP. We found that gastrodin activated DAF-16 and induced DAF-16 nuclear translocation after 24 h treated with gastrodin (*P* < 0.0001, *F* = 3.035, *df* = 18; Fig. 3d, e). These results indicated that gastrodin functions via DAF-16 to counter the symptoms of Parkinson’s disease.

We next asked whether DAF-2 acted upstream of DAF-16 to confer anti-Parkinson properties. Compared with the wild type strain (N2), the *daf*-*2* mutant exhibited significantly increased chemotaxis (*P* = 0.0078 < 0.01, *F* = 2.351, *df* = 4; Fig. 3f). Moreover, the double mutant *daf*-*2(e1370);daf*-*16(mgDf47)* obviously rescued the chemotaxis index to the level of wild type animals (*P* = 0.0327 < 0.05, *F* = 1.856, *df* = 4; Fig. 3f). Together, our data preliminarily confirmed that gastrodin could act to protect from Parkinson’s disease via the DAF-2/DAF-16 signaling pathway.

### DAF-2 is required for neuroprotective effect of dopamine neuron in PD

**Fig. 4:**
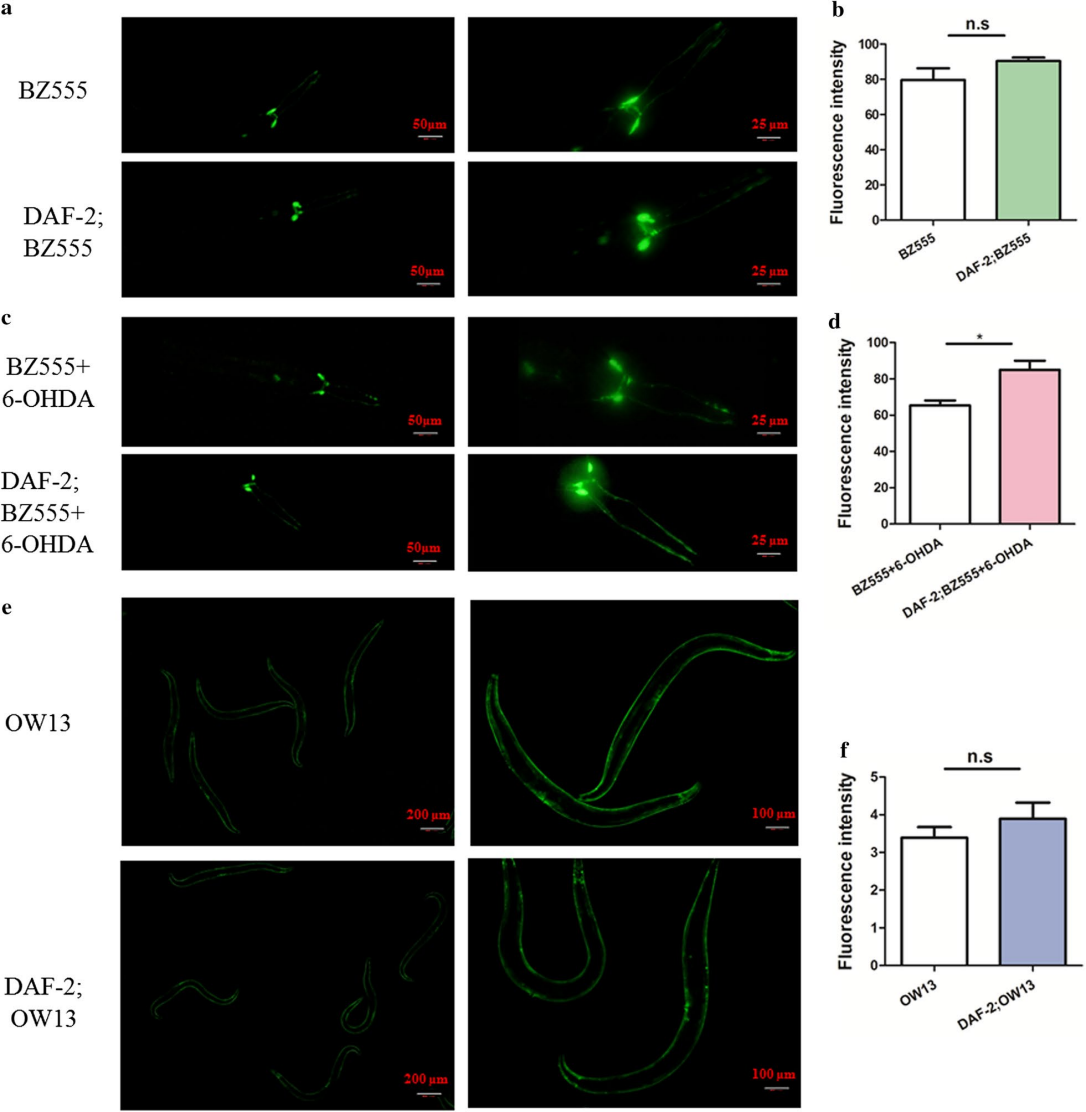
DAF-2 is involved in the protection role of dopamine neurons in PD. **a** The expression of BZ555 *egIs1 [dat*-*1p::GFP]* was similar as the expression in *daf*-*2(e1370)* mutants. The scale bar and magnification of left panel is 50 μm, 200; The scale bar and magnification of right panel is 25 μm, 400, respectively. **b** Quantification of *dat*-*1p::GFP* was no changed in wild type and *daf*-*2(e1370)* mutant animals. **c** *daf*-*2* mutants were more resistant to the injury of 6-OHDA treatment. The scale bar and magnification of left panel is 50 μm, 200; The scale bar and magnification of right panel is 25 μm, 400, respectively. **d** Quantification of *dat*-*1p::GFP* animals was induced than that of *daf*-*2(e1370)* mutants treated with 6-OHDA. **e** DAF-2 is not required for the accumulation of α-synuclein. (The scale bar and magnification of left panel is 200 μm, 40; The scale bar and magnification of right panel is 100 μm, 100). **f** Quantification of α-synuclein in wild type was consistent with that in *daf*-*2(e1370)* mutant animals. The statistical differences were analyzed using *t*-test, ns *P* ≥ 0.05; *P < 0.05; **P < 0.01; ***P < 0.001

The above data suggest a role of the insulin-like pathway in PD, and we next used a genetic method to test if insulin-like signaling could protect dopamine neurons from degeneration. To obtain the double mutant, we crossed BZ555 [*egIs1 [dat*-*1p::* GFP*]*] with the *daf*-*2* mutant. Then, it was found that the GFP signal corresponding to dopamine neurons in the *daf*-*2(e1270)* mutant was unchanged relative to the double mutant *daf*-*2(e1370); egIs1[dat*-*1p::GFP]* (*P* = 0.1966 > 0.05, *F* = 11.4, *df* = 4; Fig. 4a, b). However, we observed a change in the level of GFP in response to treatment with 6-OHDA in wild type worms and *daf*-*2* mutant worms. As shown in Fig. 4c, d, BZ555 animals showed decreased GFP expression in dopamine neurons, and the *daf*-*2* mutant significantly resisted 6-OHDA treatment compared with wild type worms (*P* = 0.029 < 0.05, *F* = 3.42, *df* = 4; Fig. 4c, d). When we crossed OW13 [*grk*-*1(ok1239) X; pkIs2386 IV*] with the *daf*-*2* mutant, we found no change in the accumulation of α-synuclein in the *daf*-*2(e1270);* grk-1(ok1239) X; pkIs2386 IV (*P* = 0.3464 > 0.05, *F* = 2.29, *df* = 12; Fig. 4e, f). Our results further suggest that DAF-2 only mediated the activity of dopamine neurons not α-synuclein. All of the data indicate that gastrodin may alleviate dopamine neuron injury and α-synuclein accumulation, and mediates neuroprotection of dopaminergic neurons by the DAF-2/DAF-16 insulin-like pathway in the PD model.

In conclusion, we found that gastrodin regulated the DAF-2/DAF-16 pathway to repair the injury of dopamine neurons but not to reduce α-synuclein accumulation in PD model. Therefore, the decreased levels of α-synuclein protein resulting from gastrodin treatment are likely mediated by other pathways in the PD model.

## Discussion

Gastrodin has been extensively studied in the treatment of neurodegenerative disease, but its mechanism remains to be elucidated. Our results indicate that gastrodin may attenuate dopamine neuron injury and α-synuclein accumulation, and mediates neuroprotection of dopaminergic neurons via the DAF-2/DAF-16 insulin-like pathway in the PD model. Gastrodin mediates oxidative stress, ERK1/2 and GSK-3β pathways, apoptosis, and p38 MAPK/Nrf2 pathway to protect dopamine neurons in several PD models [14–17]. But, up to now, there are little reports that gastrodin alleviate PD through interfering with insulin pathway. Numberous studies have highlighted that insulin signaling is involved in neurodegeneration [26–28]. Likewise in *C. elegans* PD model, insulin-IGF-1 receptor DAF-2 mutation eases α-synuclein aggregation, resist more different stress [27]. Morris and co-workers demonstrate that insulin resistance could damage the dopaminergic neurons via increased iron accumulation [36]. It is reported that IGF-1/insulin-like signaling modulates dopamine neurodegeneration induced by α-synuclein independent of DAF-16/FOXO in *Drosophila* and *C. elegans* models [37]. Our study also found that DAF-2 is a key gene in PD model treated with gastrodin, however, DAF-16 which is a transcription factor acts downstream of DAF-2 to resist injury of dopamine neurons in *C. elegans* PD model.

Gastrodin still has effect on other central nervous system disease, including epilepsy, Alzheimer’s disease, and even affective disorders, cognitive impairment. Gastrodin suppresses protein MAPK-associated inflammatory response, apoptosis in epilepsy [38, 39]. Studies have shown that pretreatment of gastrodin could anti-inflammatory, anti-oxidative, anti-apoptotic an so on for decreasing the production and aggregation of Aβ against Alzheimer’s disease [40–42]. Based on previous reports, DAF-2 refers to aging, glucose metabolism, toxic protein accumulation [26, 37]. And, gastrodin can modulate DAF-2/DAF-16 in our result. Hence, it is speculated that gastrodin may delay aging, improve metabolism and reduce proteotoxicity to prevent other neurodegenerative disease by insulin-like signaling.

Many patients with Parkinson’s disease exhibit non-motor symptoms of olfaction dysfunction, but olfaction assessment is not a clinical diagnosis. Ethanol preference and nonanol repulsion assay has been reported to be used as a chemotaxis behavior phenotypes in PD models of *C. elegans* [43, 44]. From our data, it was found that chemotaxis behavior to 2-heptanone was an indicator of olfaction dysfunction in the *C. elegans* PD model. However, chemotaxis behavior to benzaldehyde was unaffected in our assay (data not shown). The responses to 2-heptanone and benzaldehyde depend on AWC chemosensory neurons, but the downstream signaling of the two odorants is different, involving G-protein-coupled receptors (GPCRs) and G protein, respectively [45, 46]. Hence, we suggest that dopamine neuron damage and α-synuclein accumulation do not result in the olfactory dysfunction of AWC neurons. The chemotaxis assasy to 2-heptanone in PD model could be a damaged downstream signaling pathway of AWC chemosensory neurons. But, we advised that the chemotaxis behavior to 2-heptanone might be an indicator in PD model. In addition, after treatment with gastrodin, the chemotaxis index of the *daf*-*2(e1370)* mutant was still obviously higher than for animals without gastrodin as shown in Fig. 3f. Thus, gastrodin may act on an additional signal pathway to activate DAF-16 to protect against Parkinson’s disease.

## Conclusions

Here, we found that gastrodin regulated the DAF-2/DAF-16 pathway to repair the injury of dopamine neurons but not to reduce α-synuclein accumulation in the *C. elegans* PD model. Therefore, the decreased levels of α-synuclein protein resulting from gastrodin treatment are likely mediated by other pathways in the PD model.

## Methods

### Strains of *C. elegans*

The *C. elegans* strains used in this experiment were obtained from Caenorhabditis Genetics Center in Table 1. Nematodes were maintained and grown on nematode growth media (NGM) plates with *Escherichia coli* strain OP50 culture at 20 °C according to the standard protocol [47].

**Table 1:** The strains used in the study

Name	Genotype	Description
N2	*C. elegans* wild isolate	*C. elegans* var Bristol
BZ555	egIs1	egIs1 [dat-1p::GFP]
OW13	grk-1(ok1239)X; pkIs2386 IV	pkIs2386[unc-54p::alpha-synnuclein::YFP + unc-119(+)]
CB1370	daf-2(e1370) III	daf-2(e1370) III
GR1309	daf-16(mgDf47)I; daf-2(e1370) III	mgDf47 completely suppresses daf-c phenotype of daf-2. mgDf47 deletes approximately 8 kb of the daf-16 gene beginning after exon 4
CF1038	daf-16(mu86) I	Dauer defective. Short lived
TJ356	zIs356 IV	zIs356[daf-16p::daf-16a/b::GFP + rol-6(su1006)]

### Screening right concentration of gastrodin assay

Gastrodin was obtained from Sigma-Aldrich (SMB00313, USA). The gastrodin was dissolved with ddH_2_O for 5 mM. Then, different volume gastrodin was diluted in NGM medium to different concentraions (25, 50, 100, 200 μM). The right concentration of gastrodin assay was evaluated by food clearance assay and pumping rate assay. The different concentrations (0, 25, 50, 100, or 200 μM) of gastrodin was added in NGM or S medium and determined by monitoring the food clearance and the pumping rate.

In the food clearance assay, the same OD (optical density) of freshly incubated OP50 culture, about 35 L1 wild type worms and five different concentrations gastrodin were added to 96-well plates having S medium. The plates containing *C. elegans* and OP50 culture were placed in 25 °C, then the OD595 nm of OP50 was determined by Microplate Reader daily.

Pumping rate was calculated by using microscopy, assessed on OP50/NGM plates. The pumping rate means the pharyngeal movement of worms. At the beginning of the test, thirty L4 worms were transferred onto each plate with a worm picker. After 24 h, the pumping rate was determined for 10 individuals, located inside the bacterial lawn, by counting the pharynx grinder movements within a 30 s period.

### Behavior analysis

In our assay, the olfactory dysfunction of PD model animals was assayed by measuring the chemotaxis response of animals to 2-heptanone (537683, Sigma-Aldrich, USA). Based on the reports by Bargmann, the chemotaxis index assay was performed as described previously in 10 cm culture plates containing 10 ml of 1.6% agar [48]. There were two marks on the back at 0.5 cm edge of the assay plates symmetrically. Then, 1 μl 10^−1^ 2-heptanone (diluted in ethanol) was dropped in one mark, and 1 μl control reagent ethanol was dropped in the other symmetrical mark. Then, the well-fed animals without bacteria after washed using M9 buffer were put in the center of plates. The numbers of worms at the two sides were counted respectively after 1 h. The chemotaxis index was scored using the formula = worms numbers at 2-heptanone side - worms numbers at control side/total animal number. The behavior assay had been repeated three times with 30-40 worms per assay.

### *C. elegans* model of PD and Gastrodin treatment

We established PD model by 6-Hydroxydopamine (6-OHDA) according to Nass’s report [49]. About 150 L3 stage worms were harvest, washed with M9 buffer, and added to a mixture including 10 mM ascorbic acid and 50 mM 6-OHDA. The worms were treated for 1 h at 24 °C, mixed mildly every 15 min, washed with M9 buffer. After the PD model was successfully established, the animals were divided into two groups including PD model group and gastrodin group. The animals of PD model group were laid in OP50/NGM medium addition with no gastrodin for 24 h, then treated with floxuridine (purchased from Sigma, F0503) in 22 °C; The animals of gastrodin group were laid in OP50/NGM medium addition with gastrodin for 24 h, then treated with gastrodin and floxuridine in 22 °C. Both of the control group and gastrodin group were observed and further test after 72 h in Fig. 1a. Every experiment was performed at least three independent replicates.

### Crossing worms

We obtained double mutants by crossing techniques [50]. The crosses were done by 6-10 males and 2 L4 stage hermaphrodites, and then the homozygous progeny was screened by Polymerase Chain Reaction (PCR) assay. After F1 produced, if there were male and female worms with fluorescent marker, the F1 generation is crossed successfully. Further, we also cloned a 602 bp daf-2 fragment for verify crossed F1 generation using primers CATCAAGATCCAGTGCTTCTG and GACGGACTGCA

-ATTTTTCG. Then, we allow F1 generation to self-cross for screening the homozygous progeny. We still cloned DAF-2 gene by PCR. Due to only one base mutation in *daf*-*2(e1370)* mutant, we sequenced the double stranded PCR product to obtain homozygous crossed worms.

### Quantifying of fluorescence

The two PD models were treated with or without gastrodin as the above methods. The fluorescence of dopaminergic neurons and α-synuclein expression was observed using Olympus BX51 fluorescence microscopy (Tokyo, Japan) after 24 h, 48 h, 72 h. The fluorescence intensity of dopaminergic neurons and α-synuclein was obtained by Image J software. The mean fluorescence intensity was measured at least three animals.

### DAF-16:: GFP expression analysis

DAF-16::GFP worms in L3 stage were treated with gastrodin or no gastrodin in NGM plates having floxuridine. After 24 h, 48 h, 72 h, the distribution of DAF-16::GFP was observed using Olympus BX51 fluorescence microscopy (Tokyo, Japan). The nuclear location of DAF-16::GFP was counted by the accumulation of GFP in the nuclear.

### Determination of dopamine content

The content of dopamine were performed by a high-performance liquid chromatography system (LC-30A, Shimadzu, Japan) coupled with tandem mass spectrometer (API 3200, AB Sciex, USA).The synchronized worms were washed with M9 buffer to remove bacteria, harvested in 500 µl 0.2 M HClO_4_, and then sonicated and centrifuged at 12000 rpm for 15 min. The supernatants were prepared for quantification of dopamine.

### Statistical analysis

All data were analyzed using GraphPad Prism. no significant (ns) *P* > 0.05; *P < 0.05, **P < 0.01, ***P < 0.001. Three independent trails were performed.

## Additional files

**Additional file 1: Fig. S1**. The dopamine neurons of worms treated with gastrodin is no changed. The fluorescence intensity of BZ555 with gastrodin was similar to that of worms without gastrodin using *t* test, ns *P* ≥ 0.05, *P < 0.05, **P < 0.01, ***P < 0.001.

**Additional file 2: Fig. S2**. DAF-16 increased the content of dopamine by LC–MS/MS. (A) The content of dopamine in wild type worms and *daf*-*16(mu86)* mutant treated with gastrodin using LC–MS/MS. (B) Quantification of wild type worms and *daf*-*16(mu86)* mutant having gastrodin or no gastrodin by *t*-test, ns *P* ≥ 0.05, *P < 0.05, **P < 0.01, ***P < 0.001.

## Data Availability

The datasets used or analysed during the current study are available from the corresponding author on reasonable request.

## Abbreviations

PD: Parkinson’s disease
TCM: traditional Chinese medicine
*C. elegans*: *Caenorhabditis elegans*
6-OHDA: 6-Hydroxydopamine
MAPK: mitogen-activated protein kinases
PCR: Polymerase Chain Reaction
NGM: nematode growth media
OD: optical density
GFP: green fluorescent protein
GPCRs: G-protein-coupled receptors
