# Supplementary figures and images for: Gastrodin protects dopaminergic neurons via insulin-like pathway in a Parkinson’s disease model
Source: BMC Neurosci. 2019 Jun 17;20:31. doi: 10.1186/s12868-019-0512-x (PMC6580469; doi:10.1186/s12868-019-0512-x)

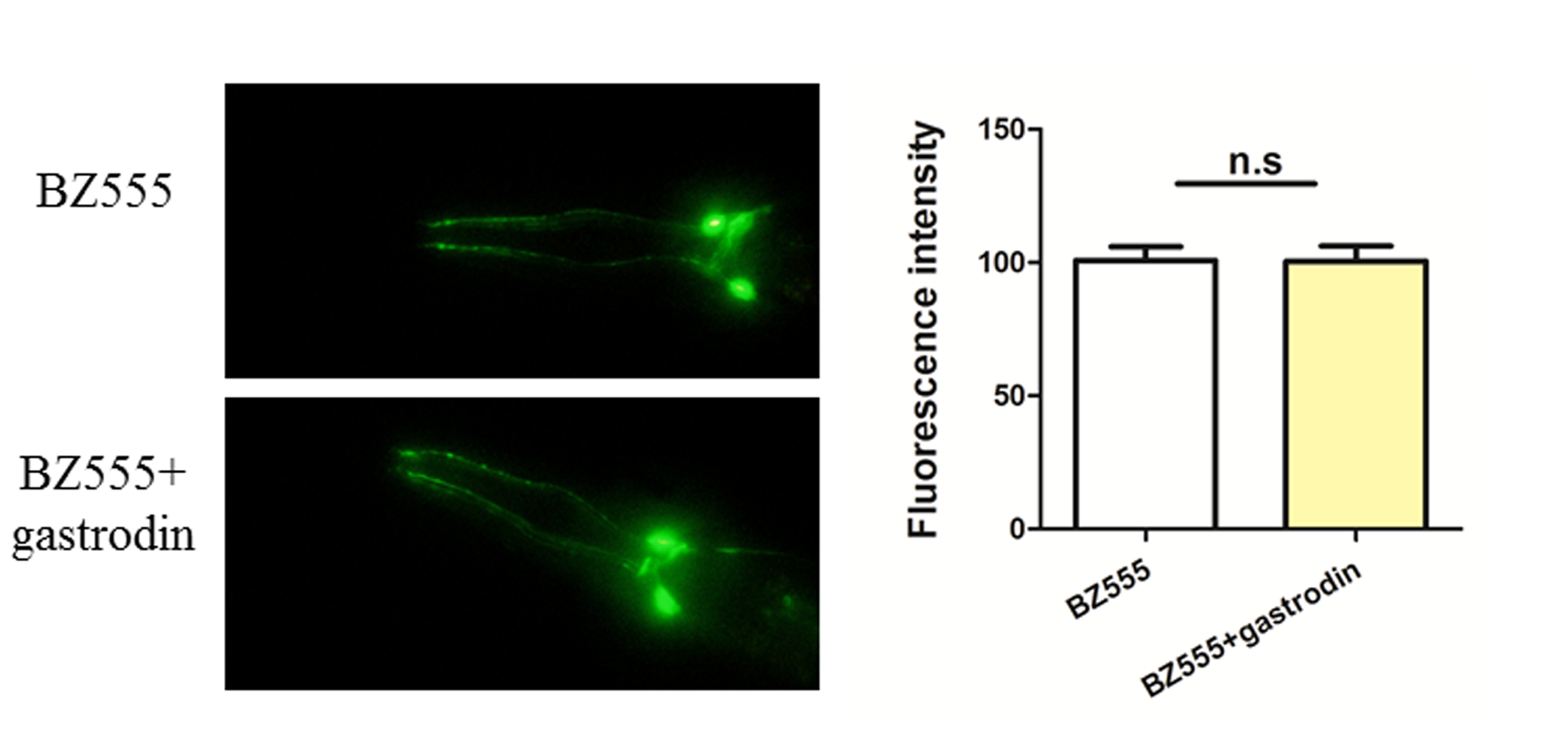

Supplement: Supplementary file 1 — Additional file 1: Fig. S1. The dopamine neurons of worms treated with gastrodin is no changed. The fluorescence intensity of BZ555 with gastrodin was similar to that of worms without gastrodin using t test, ns P ≥ 0.05, *P < 0.05, **P < 0.01, ***P < 0.001. [file 12868_2019_512_MOESM1_ESM.tif]

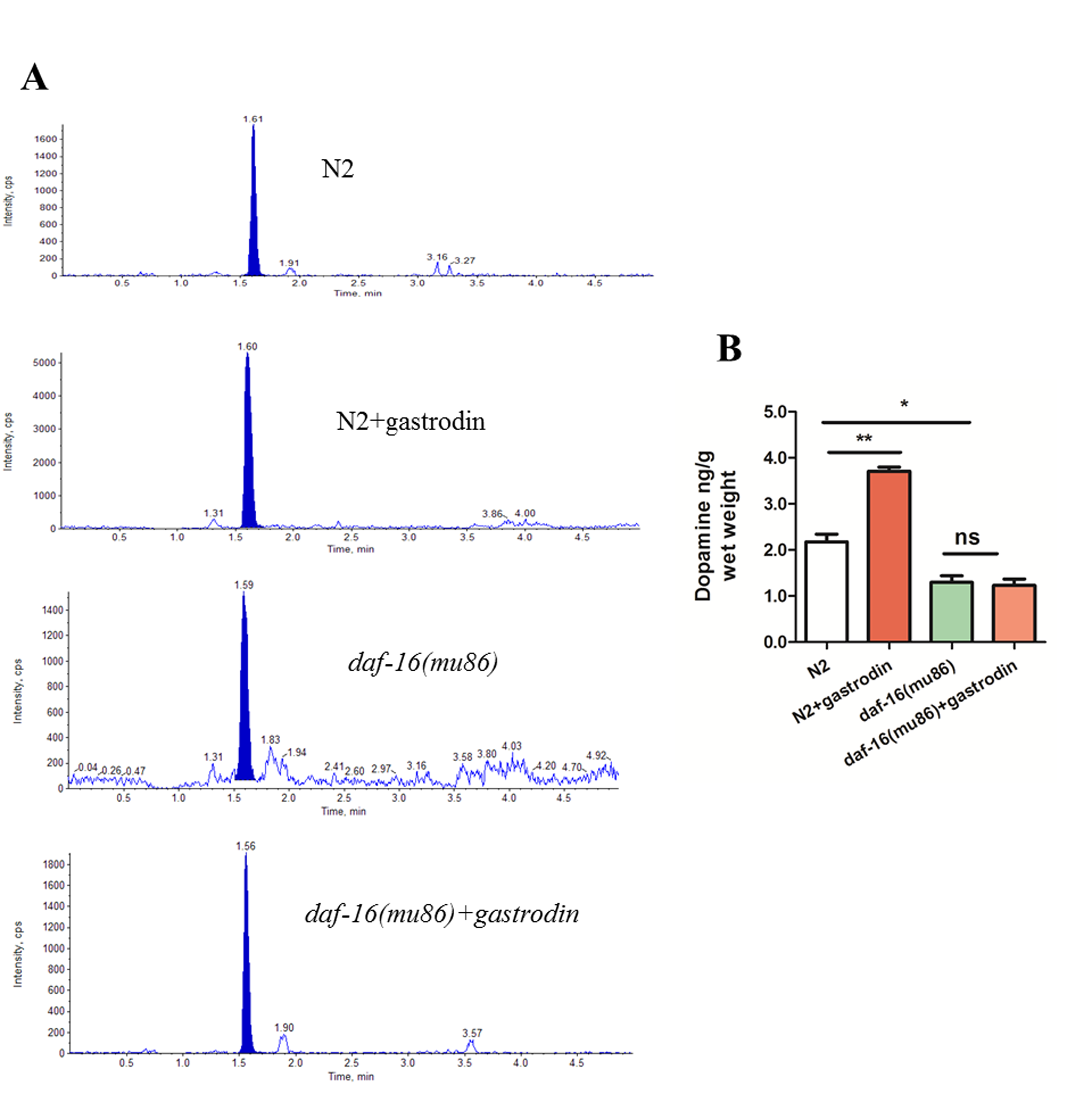

Supplement: Supplementary file 2 — Additional file 2: Fig. S2. DAF-16 increased the content of dopamine by LC–MS/MS. (A) The content of dopamine in wild type worms and daf-16(mu86) mutant treated with gastrodin using LC–MS/MS. (B) Quantification of wild type worms and daf-16(mu86) mutant having gastrodin or no gastrodin by t-test, ns P ≥ 0.05, *P < 0.05, **P < 0.01, ***P < 0.001. [file 12868_2019_512_MOESM2_ESM.tif]
